# Cost-Effectiveness Analysis of Fourth- or Further-Line Ripretinib in Advanced Gastrointestinal Stromal Tumors

**DOI:** 10.3389/fonc.2021.692005

**Published:** 2021-12-06

**Authors:** Weiting Liao, Huiqiong Xu, David Hutton, Qiuji Wu, Kexun Zhou, Hui Luo, Wanting Lei, Mingyang Feng, Yang Yang, Feng Wen, Qiu Li

**Affiliations:** ^1^ Department of Medical Oncology, Cancer Center, West China Hospital, Sichuan University, Chengdu, China; ^2^ West China Biomedical Big Data Center, Sichuan University, Chengdu, China; ^3^ Department of Health Management and Policy, University of Michigan, Ann Arbor, MI, United States; ^4^ State Key Laboratory of Biotherapy and Cancer Center, West China Hospital, Sichuan University and Collaborative Innovation Center, Chengdu, China

**Keywords:** gastrointestinal stromal tumor, resistant, ripretinib, cost-effectiveness, fourth-line or further

## Abstract

**Background:**

The INVICTUS trial assessed the efficacy and safety of ripretinib compared with placebo in the management of advanced gastrointestinal stromal tumors.

**Method:**

We used a Markov model with three health states: progression-free disease, progression disease and death. We parameterized the model from time-to-event data (progression-free survival, overall survival) of ripretinib and placebo arms in the INVICTUS trial and extrapolated to a patient’s lifetime horizon. Estimates of health state utilities and costs were based on clinical trial data and the published literature. The outcomes of this model were measured in quality-adjusted life-years (QALYs), costs, and incremental cost-effectiveness ratios (ICERs). Uncertainty was tested *via* univariate and probabilistic sensitivity analyses.

**Results:**

The base-case model projected improved outcomes (by 0.29 QALYs) and additional costs (by $70,251) and yielded an ICER of $244,010/QALY gained for ripretinib versus placebo. The results were most sensitive to progression rates, the price of ripretinib, and health state utilities. The ICER was most sensitive to overall survival. When overall survival in the placebo group was lower, the ICER dropped to $127,399/QALY. The ICER dropped to $150,000/QALY when the monthly cost of ripretinib decreased to $14,057. Probabilistic sensitivity analyses revealed that ripretinib was the cost-effective therapy in 41.1% of simulations at the willingness-to-pay (WTP) threshold of $150,000.

**Conclusion:**

As the fourth- or further-line therapy in advanced gastrointestinal stromal tumors, ripretinib is not cost-effective in the US. Ripretinib would achieve its cost-effectiveness with a price discount of 56% given the present effectiveness.

## Introduction

Gastrointestinal stromal tumors (GISTs) are the most common mesenchymal neoplasms of the digestive tract and can be located in the stomach (60%), small intestine (30%), duodenum (4-5%), rectum (4%), colon and appendix (1-2%), and esophagus (<1%), and rarely as apparent primary extra gastrointestinal tumors in the vicinity of the stomach or intestines (1). Approximately 95% of GISTs contain pathogenic mutations in one of two tyrosine kinase receptor genes: KIT and PDGFRA (2).

Imatinib mesilate, an oral tyrosine inhibitor with activity against KIT and PDGFRA (3), remains the standard first-line therapy for patients with metastatic or unresectable GIST. However, in the extended follow-up of the pivotal B2222 study, 5% of patients showed primary resistance within the first two months (4), and second or acquired resistance developed after a median of approximately 2 years of treatment with imatinib (5). In the setting of imatinib failure, another TKI sunitinib malate with selectivity for KIT and PDGFRA (6) brought a mean time-to-progression of approximately 7 months (7), resulting in approval of sunitinib as the second-line therapy. Then, third-line regorafenib (8) showed significant improvement in progression-free survival (PFS) compared with placebo (4.8 months versus 0.9 months) for patients with previous failure of at least imatinib and sunitinib. The progression mechanism is mainly summarized by the development of secondary resistance mutations in the ATP binding domain or activation loop of KIT/PDGFRA (9).

Ripretinib acts as a novel type II tyrosine switch control inhibitor to broadly inhibit drug-resistant mutations in KIT and PDGFRA (10). In the INVICTUS trial (11), the median overall survival(OS) was 15.1 months in the ripretinib group versus 6.6 months in the placebo group (HR 0.36, 95% CI 0.21-0.62). Ripretinib had an acceptable safety profile, with mainly low-grade and controllable adverse effects. The US Food and Drug Administration first granted ripretinib (QINLOCK) approval for adult patients with advanced GIST who have received prior treatment with ≥ 3 kinase inhibitors on 15 May 2020 (12). The advent of ripretinib indicates a major advance for the therapy of advanced GIST. Likewise, it is potential to tremendously add costs and influence health care budgets. This increased expenses must be weighed against the long-term benefits to make an informed decision targeting this disease in clinical practice.

As the cost of imatinib eventually declines with the availability of generic imatinib, and the reported monthly ripretinib treatment cost of $32,000, a thorough evaluation of whether the increased clinical benefit outweighs the cost is warranted (13). In this analysis, we aimed to project the potential cost-effectiveness of fourth-line (or more) ripretinib for patients with advanced GIST from the perspective of the US payer.

## Methods

### Model Structure

**Figure 1:**
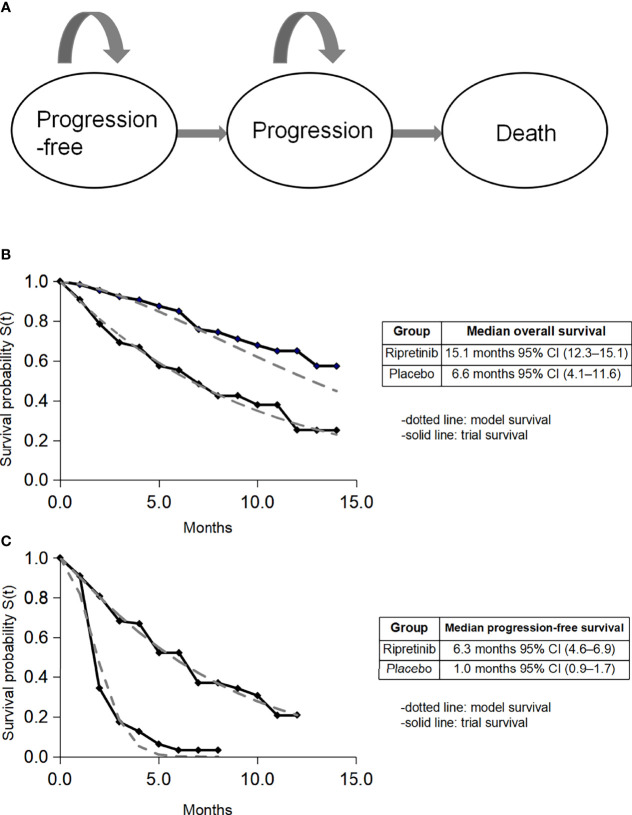
Model construction, survival data, and fitted survival data. CI, confidence interval. **(A)** Model diagram; **(B)** fitted overall survival; **(C)** fitted progression-free survival.

We developed a Markov model through clinical data from the INVICTUS randomized clinical trial containing three mutually exclusive health states: progression-free disease, progression disease, and death (**Figure 1A**). This model compared two strategies for treating patients with advanced GIST: (1) ripretinib plus best supportive care (BSC) and (2) BSC. A discount rate of 3% per annum was used for costs and health benefits, and a half-cycle correction was included. As many US-based cost-effectiveness analyses focus on the payer’s decision regarding the coverage and reimbursement of health care (14), this analysis took the payer’s perspective.

### Clinical Data, Costs and Quality of Life

Eligible patients had progression on at least imatinib, sunitinib, and regorafenib, or documented intolerance to any of these therapies despite dose modifications, then they were assigned to receive either oral ripretinib 150 mg plus BSC once a day or BSC for 28-day cycles (11). Individual patient data on PFS and OS within the treatment arm were extracted using GetData Graph Digitizer (http://getdata-graph-digitizer.com/). Event time distributions were estimated using the flexible parametric Weibull model for the tail in both immediate and delayed ripretinib arms. The fitted survival curves are shown in **Figures 1B, C**.

The main input parameters are shown in **Table 1**. The costs related to healthcare services were inflated to 2021 values based on the US Consumer Price Index (15). For the progression-free status, we included drug price, cost for adverse management and supportive care. According to Redbook, the list price of ripretinib was $32,000 per 30 doses (16). The cost for BSC was $3,382.47 per month (17). The cost for the management of adverse events was $421.2, and the cost for computed tomography was $1,365.39 per time (17). Total costs for grade 3 to 5 complications were calculated using the frequency for each adverse event multiplied by the cost of adverse effects per event. For costs of progressive disease, we calculated the cost of BSC in the ripretinib group, while for the alternative delayed treatment group, we included the cost of BSC and added the cost of ripretinib for the proportion (29 of 44) that crossed over to ripretinib in the INVICTUS trial (11). The trial survival data indicated placebo patients who crossed over after progression gained survival benefits, in terms of both PFS and OS, which are reflected in the Kaplan-Meier curves (11).

**Table 1:** Input data for the Markov model in patients with gastrointestinal stromal carcinoma.

Variable	Ripretinib	Placebo
***Monthly Costs ($)***		
Repretinib	32,000	0
Best supportive care	3,382.47	3,382.47
Adverse events per occurrence	421.2	421.2
Computed tomography per time	1,365.39	1,365.39
***Utility***		
PF	0.767	0.767
PD	0.647	0.647
Death	0	0
***Weibull OS model***		
intercept	2.782163	2.250612
log_scale	-0.4285523	0.00098579
gamma	1.535033646	0.999014696
lambda	0.013971806	0.105568583
***Weibull PFS model***		
intercept	2.074947	0.830945
log_scale	-0.09223515	-0.6655104
gamma	1.096622663	1.945483243
lambda	0.102752314	0.19857477

PF, progression-free; PD, progression disease; OS, overall survival; PFS, progression-free survival.

In terms of health-related quality-of-life measures, the utilities of progression-free disease and progression disease were estimated according to published utilities equal to 0.767 for progression-free disease and 0.647 for progression disease (18).

### Sensitivity Analyses

One-way sensitivity analyses are shown in tornado diagrams within the appropriate ranges. Probabilistic sensitivity analysis using 10,000 Monte Carlo simulations was performed to further address the uncertainty of the results, using gamma distributions for cost parameters, beta distributions for utilities, and normal distributions for Weibull survival parameters (19, 20).

### Cost-Threshold Analysis

A cost-threshold analysis was performed to determine the cost of ripretinib at which it would become cost-effective as a fourth- or further-line therapy.

### Statistical Analyses

The Markov model was developed with TreeAge Pro (Williamstown, MA), and additional statistical analyses were conducted with R version 3.6.3. The outcomes of this model were measured in quality-adjusted life-years (QALYs), costs, and incremental cost-effectiveness ratio (ICER). The proposed treatment is deemed “cost-effective” if the ICER is below a willingness-to-pay (WTP) threshold of $150,000 (21–23).

## Results

### Base-Case Analysis

In the base-case scenario, placebo generated a total cost of $189,854 and a total 0.52 QALYs, while ripretinib generated a total cost of $260,105 and a total 0.81 QALYs, with an ICER of $244,010 per QALY in the patients with advanced GIST (**Table 2**). The cost-effectiveness results without rounding were shown in **Supplementary Table**.

**Table 2:** Results for estimated costs and consequences.

Variance	Ripretinib	Placebo
***Cost ($)***		
Cost for PF state	234,808	9,505
Cost for PD state	25,297	180,349
Total cost	260,105	189,854
Incremental cost	70,251	
***Effectiveness (QALYs)***		
Effectiveness for PF state	0.41	0.13
Effectiveness for PD state	0.40	0.39
Total effectiveness	0.81	0.52
Incremental effectiveness	0.29	
***Incremental cost-effectiveness ratio ($/QALY)***	244,010	

PF, progression-free; PD, progression disease; QALY, quality-adjusted life-year.

### Sensitivity Analyses

**Figure 2:**
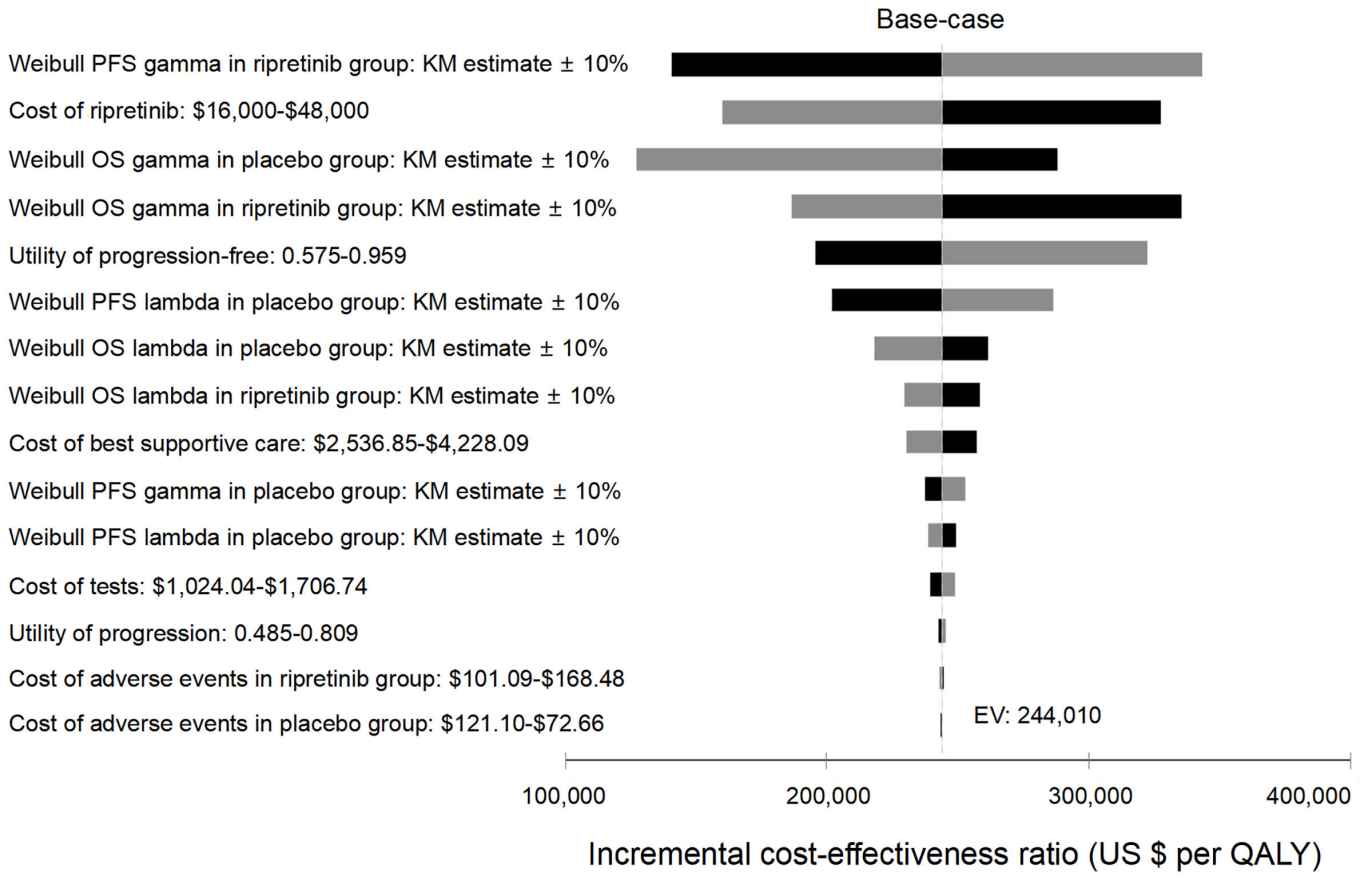
Tornado analysis demonstrating results from one-way sensitivity analysis. Kaplan-Meier, KM; QALY, quality-adjusted life-year; OS, overall survival; PFS, progression-free survival.

One-way deterministic sensitivity analyses revealed that the most influential variables that altered the cost-effectiveness of the strategies were PFS progression rates, the price of ripretinib, OS progression and health state utilities (**Figure 2**). The lower limit of ICER was most sensitive to Weibull OS gamma in the placebo group. When the weibull gamma relating to the OS in the placebo group became lower (0.89911) indicating faster mortality in the placebo group, the ICER dropped to $127,399/QALY. One-way sensitivity analysis on monthly cost of ripretinib found that the ICER was $160,178/QALY and $143,412/QALY if the price of ripretinib was decreased to $16,000 (50% off the price) or $12,800 (60% off the price) per month, respectively (**Table 3**).

**Table 3:** One-way sensitivity analysis on monthly cost of ripretinib.

Cost of ripretinib per month, $	Total Cost, $	Effectiveness, QALYs	Incremental cost, $ (compared with placebo)	Incremental effectiveness, QALYs (compared with placebo)	ICER, $/QALY
					(compared with placebo)
3,200	76,754	0.81	26,807	0.29	93,113
(90% discount)					
6,400	97,127	0.81	31,634	0.29	109,879
(80% discount)					
9,600	117,499	0.81	36,461	0.29	126,645
(70% discount)					
12,800	137,871	0.81	41,288	0.29	143,412
(60% discount)					
16,000	158,244	0.81	46,115	0.29	160,178
(50% discount)					
19,200	178,616	0.81	50,942	0.29	176,944
(40% discount)					
22,400	198,988	0.81	55,769	0.29	193,711
(30% discount)					
25,600	219,361	0.81	60,596	0.29	210,477
(20% discount)					
28,800	239,733	0.81	65,424	0.29	227,243
(10% discount)					
32,000*	260,105	0.81	70,251	0.29	244,010

ICER, incremental cost-effectiveness ratio; QALY, quality-adjusted life-year; WTP, willingness-to-pay.

*Current price.

**Figure 3:**
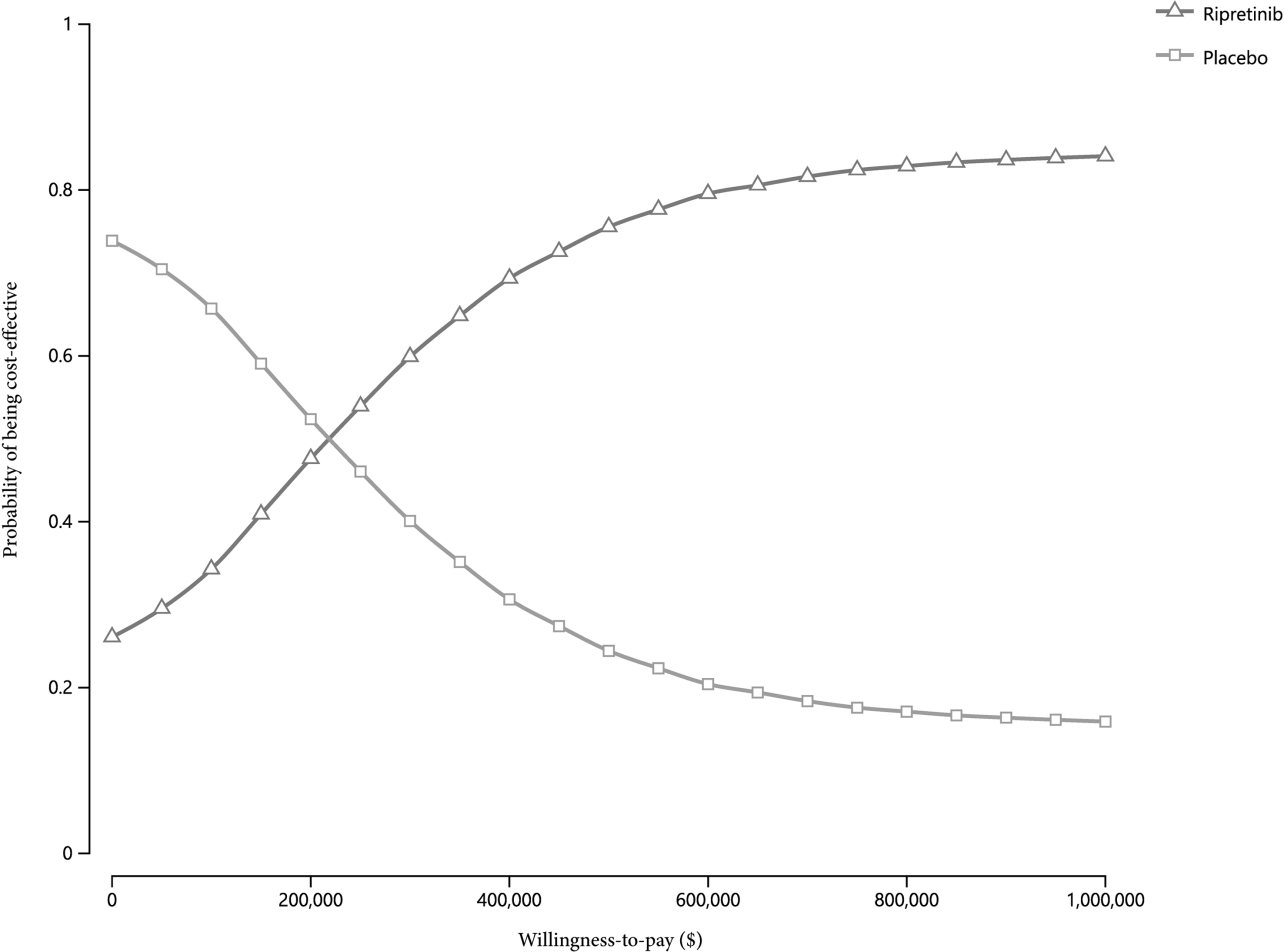
Cost-effectiveness acceptability curves of ripretinib versus placebo across a range of cost-effectiveness ratios.

The cost-effectiveness acceptability curves revealed that ripretinib was the cost-effective therapy in 41.1% of 10,000 simulations given the present price of ripretinib at the cost-effectiveness threshold of $150,000/QALY (**Figure 3**). Moreover, ripretinib reached a 48.5% probability of being cost-effective at the WTP threshold of $200,000/QALY.

### Cost-Threshold Analysis

In the threshold analysis for ripretinib to be cost-effective compared with placebo based on the WTP level of $150,000, the listed price of ripretinib would have to be reduced by 56% to $14,057 per month (approximately $468.6 per dose).

## Discussion

The approval of fourth- or further-line ripretinib for GIST offers an effective treatment alternative to forestall progression. We found immediate ripretinib therapy generated 0.29 QALYs at an incremental cost of $70,251, for an ICER of $244,010 per QALY compared with placebo in the patients with advanced gastrointestinal stromal carcinoma. Ripretinib could become cost-effective ($150,000/QALY) by reducing the price by approximately 56% from its current price of $32,000 per month.

Since ripretinib was approved recently (12), modeling studies like this one can combine the latest data to give informative evaluations (24) in the absence of trial-based cost-effectiveness analyses. Current cost-effective analyses of GIST in US or non-US countries are relatively limited, with only a few studies exploring the cost-effectiveness of previously approved tyrosine kinase inhibitors. Based on the US data, imatinib mesylate therapy for unresectable GIST (total cost: $416,255 over 10-year horizon) increased 1.90 QALYs at a marginal cost of $74,369, producing an ICER of $38,723 per QALY (25). From the perspective of the national health payer in Mexico, high-dose imatinib as second-line treatment had a mean cost of $35,225, whereas sunitinib had a mean cost of $17,805 (26). In the setting of Germany, regorafenib treatment (total cost: €22,102) provided 0.42 QALYs versus imatinib rechallenge (total cost: €13,329) over a lifetime horizon, which produced an ICER of €21,127 per QALY gained (18). According to the perspective of the Spanish National Health System, sunitinib (total cost: €23,259) as second-line treatment projected to have 1.00 QALYs in metastatic and/or unresectable GIST, while BSC (total cost: €1,622) has 0.55 QALYs, with the ICERs of €49,090 per QALY (27). In this analysis, the total cost of the fourth-line ripretinib was $260,105 over a lifetime horizon, higher than reported costs of most frontier therapies except imatinib, which could be attributed to the long-term survival brought by ripretinib and continuous treatment until the course of the disease progresses. Overall, the accumulated cost of treatment over the duration of therapy poses a heavy burden on GIST patients who advance to late stages.

Our study is the first cost-effectiveness analysis of ripretinib, designated orphan drug for the fourth- or further-line treatment in GIST. The conclusion in this analysis that it yields substantial health gains but is not cost-effective reflects the high price of ripretinib therapy. The current challenge faced by patients is lack of appropriate access to these orphan drugs while acquiring value from drug spending (28). We determine that ripretinib in GIST patients could be cost-effective from the perspective of a US payer with approximately 56% discounts in the cost of ripretinib. Potential measures or modifications such as putting ripretinib into more frontier-line (9) treatment and selecting patient based precise molecular typing-oriented strategy could further enhance its cost-effectiveness in GIST. Likewise, if the WTP raised to $250,000 for this ultra-rare disease with heavily pre-treated GIST, the cost-effectiveness of ripretinib may be achieved.

In a complex era where new anticancer drugs are constantly updated and fall short, the efficacy of ripretinib was commendably shown in a phase III clinical trial. Pharmaceutical companies make tremendous investments in high-risk research and development where it is difficult to find drugs that arouse clinical effects. Those efforts and cooperation of large teams and companies require the profits to guarantee the innovative investment and also to promote the vigorous development of novel drugs. Once a new medicine enters the market after approval, there are multiple aspects and benefits to be considered. However, the magnitude of clinical benefits and WTP of payers in the market should be judged, based on those benefits. Governments, policymakers and/or medical insurance companies should evaluate cost-effectiveness to understand the value and efficacy of drugs and determine reasonable coverage and discounts to ensure therapies have value.

Some limitations in this study should be noted. First, this conclusion can be comparable in countries with similar prices and treatment patterns. Given the large variability in drug costs between countries, it is necessary to use area-specific cost parameters to understand area-specific value (29). Second, the total cost in the placebo group could be higher than that in the real world because we took the crossover cost into account, which is closely related to the actual survival benefit in placebo patients. Third, the PFS and OS transition probabilities from the INVICTUS trial are uncertain. Fourth, the intangible costs are hard to be measured and the utilities were referred to previously published literature. However, these parameters were varied in deterministic sensitivity analysis. Fifth, the costs of anti-tumor drugs may decrease when generics and biosimilars come into the market or discount pricing is applied (30). Finally, we did not include the prior first through third-line treatment, because the INVICTUS trial was designed to evaluate fourth-line and beyond therapy with unknown information of the individual patients’ prior specific treatment pattern and therapeutic effects. Future cost-effectiveness analysis was expected to further assess the several potential treatment sequences rather than just comparing ripretinib with placebo in the fourth or further-line management.

In conclusion, we found that ripretinib as the fourth-line or further-line therapy for the patients in GIST was not cost-effective compared with placebo. Across the variances in the parametric distributions, the ICERs for ripretinib compared with placebo remained greater than $150,000 per QALY in most scenarios. Tornado analysis showed that the price of ripretinib was a modifiable factor that could make ripretinib cost-effective.

## Data Availability Statement

The original contributions presented in the study are included in the article/**Supplementary Material**. Further inquiries can be directed to the corresponding author.

## Author Contributions

Conception and design: QL. Data collection and assembly: WeiL, HX, DH. Data analysis and interpretation: WeiL, DH, QW, KZ. Manuscript writing: WeiL, HX. Critical revision: WeiL, HX, DH, QW, KZ, HL, WanL, MF, YY, FW, QL. All authors contributed to the article and approved the submitted version.

## Funding

The 1.3.5 project for disciplines of excellence, West China Hospital, Sichuan University (ZYJC18008, ZYJC18010) and National Natural Science Foundation of China (81802445).

## Conflict of Interest

The authors declare that the research was conducted in the absence of any commercial or financial relationships that could be construed as a potential conflict of interest.

## Publisher’s Note

All claims expressed in this article are solely those of the authors and do not necessarily represent those of their affiliated organizations, or those of the publisher, the editors and the reviewers. Any product that may be evaluated in this article, or claim that may be made by its manufacturer, is not guaranteed or endorsed by the publisher.

## Supplementary Material

Supplementary Table Cost-effectiveness results without rounding.

## Abbreviations

ICER, incremental cost-effectiveness ratio; QALYs, quality-adjusted life-years; GIST, gastrointestinal stromal tumor; WTP, willingness-to-pay; PFS, progression-free survival; OS, overall survival; QoL, quality of life; BSC, Best supportive care.
